# Cerebellar volume and functional connectivity in neonates predicts social and emotional development in toddlers

**DOI:** 10.3389/fnins.2024.1294527

**Published:** 2024-05-01

**Authors:** Jung-Hoon Kim, Kushal Kapse, Catherine Limperopoulos, Josepheen De Asis-Cruz

**Affiliations:** Developing Brain Institute, Children’s National Hospital, Washington, DC, United States

**Keywords:** structural MRI, functional MRI, multimodal study, neonate, cerebellum, ITSEA

## Abstract

Over the past decade, a growing body of research in adults has emphasized the role of the cerebellum in social and emotional cognition. This has been further supported by findings of delayed social and emotional development in toddlers with cerebellar injury during the fetal and newborn periods. However, the contributions of the cerebellum to social–emotional development in typically developing newborns are unclear. To bridge this gap in knowledge, we used multimodal MRI to investigate associations between cerebellar structure and function in 88 healthy neonates (mean ± sd of postmenstrual age, = 42.00 ± 1.91 weeks) and social–emotional development at 18-months assessed using the Infant-Toddler Social–Emotional Assessment (ITSEA) (mean age on ITSEA: 18.32 ± 1.19 months old). We found that cerebellar volume was not associated with ITSEA domain scores at 18 months. We further demonstrated cerebellar functional gradient (FGR) defined using principal component analysis (PCA) was associated with Externalizing domain (linear regression model, false-discovery-rate-adjusted *p* = 0.013). This cluster (FGR7) included the left dentate, right VI, left Vermis VIIIb, and right V lobules. Finally, we demonstrated that either structural or functional features of the cerebellum reliably predicted scores on the Externalizing and Internalizing domains (correlation between actual and predicted scores: for structural, Fisher’s *z* = 0.48 ± 0.01 for Internalizing, *p* = 0.01; for functional, Fisher’s *z* = 0.45 ± 0.01 for Externalizing, *p* = 0.02; with permutation test). Collectively, our findings suggest that the cerebellum plays an important role in social–emotional development during the critical early stages of life.

## 1 Introduction

The human cerebellum has traditionally been viewed as the controller/coordinator of motor movement (Holmes, 1939; Brodal and Bjaalie, 1997). However, research in the past decade challenges this perspective, highlighting the cerebellum’s crucial role in multisensory processing (Ishikawa et al., 2015; Schmahmann, 2019) – vermal lobules VI and VII (Stein and Glickstein, 1992), lobule VIII (Oscarsson, 1965), and lobule X (Barmack, 2003) – and higher-order cognition – lobule VI, VIIA, VIIB, and IX (Habas et al., 2009; Krienen and Buckner, 2009; O'reilly et al., 2010).

More recently, neuroimaging studies in adults have expanded our understanding of the cerebellum’s involvement in social and emotional cognition (Stoodley and Schmahmann, 2010; Van Overwalle et al., 2014; Petrosini et al., 2015). For example, Petrosini et al. (2015) reported important associations between structural variations (i.e., volume) in the posterior cerebellum region (especially in Crus I) and novelty seeking, harm avoidance, and alexithymia. Moreover, the functional properties of the cerebellum, such as event-related responses or interregional connectivity of cerebellum regions have been implicated in social decision-making (Haihambo et al., 2022), conscientiousness (Kunisato et al., 2011), and theory of mind (Van Overwalle and Vandekerckhove, 2013; Van Overwalle et al., 2015; Heleven and Van Overwalle, 2019). Collectively, these findings suggest that the cerebellum plays a key role in regulating social–emotional functioning.

Research has suggested that social attention deficits are characteristic of different neuropsychiatric disorders including autism spectrum disorder (ASD) (Baribeau et al., 2015), attention-deficit/hyperactivity disorder (ADHD) (Baribeau et al., 2015), and obsessive-compulsive disorder (OCD) (Cullen et al., 2008; Kang et al., 2012; Liu et al., 2017). In line with this, several studies have demonstrated the adverse impact of cerebellar injury during early life on social–emotional development (Bobylova et al., 2007; Limperopoulos et al., 2007; Bolduc et al., 2011). While clinical studies have significantly advanced our understanding of the mechanistic role of the cerebellum during atypical development, there remains a gap in knowledge concerning the associations between structure–function of the typically developing cerebellum and social–emotional development. This underscores the need for further research investigating how variations in cerebellar volume and functional connectivity relate to normative behavior.

In this study, we examined the association between structural and functional MRI features of the newborn cerebellum and social–emotional development at 18-months. A total of 88 MRI scans from 88 healthy, typically developing neonates were analyzed. Social and emotional development at 18-months was assessed using the Infant-Toddler Social and Emotional Assessment (ITSEA). The ITSEA is a standardized survey completed by caregivers that assesses the socio-emotional wellbeing of infants and toddlers (Carter et al., 2003). It profiles behavioral problems and competencies with scores on 4 domains: Externalizing, Internalizing, Dysregulation, and Competence (see Methods). In this normative neonatal cohort, we demonstrated that cerebellar volume was not correlated with any of the ITSEA scores. Additionally, we found that cerebellar functional gradient (FGR), or brain regions derived from data-driven clustering of functional connectivity data, was associated with Externalizing domain. We further demonstrated that scores on the Externalizing and Internalizing domains were significantly predicted by structural and functional cerebellar imaging features.

## 2 Materials and methods

### 2.1 Subjects

Participants were recruited as part of a longitudinal study investigating brain development in healthy and high-risk fetuses and newborns at Children’s National in Washington DC. Maternal exclusion criteria were psychiatric disorders, metabolic disorders, genetic disorders, complicated pregnancies, multiple pregnancies, alcohol, and tobacco use, maternal medications, and contraindications to MRI (e.g., claustrophobia). Exclusion criteria for the neonates were dysmorphic features by antenatal ultrasound, chromosomal abnormalities by amniocentesis, presentation after 28 gestational weeks, preterm birth ( ≤ 37 weeks), multiple gestation, or evidence of congenital infections. All experiments were conducted in accordance with the guidelines of the Institutional Review Board (IRB) of Children’s National. Written parental informed consent was obtained from all participants of the study.

### 2.2 Assessment of social–emotional development

Social–emotional development was assessed at 18 months using the Infant-Toddler Social and Emotional Assessment (ITSEA). The ITSEA is a previously validated, widely used survey for assessing social and emotional development in toddlers (Carter et al., 2003; Briggs-Gowan and Carter, 2006). This tool consists of four domains: Externalizing, Internalizing, Dysregulation, and Competence, each with several subscales. The Externalizing domain focuses on aggressive and impulsive behavior. The Internalizing domain evaluates depression/withdrawal, general anxiety, and separation distress. The Dysregulation domain reflects sleep quality, negative emotionality, eating behavior, and sensory sensitivity. Finally, the Competence domain assesses compliance, attention, imitation/play, motivation, and empathy. See Carter et al. (2003) and Briggs-Gowan and Carter (2006) for additional details on the ITSEA. The primary caregiver (s) rated the behavior of their child on a three-way scale: (0) not true/rarely, (1) somewhat true/sometimes, or (2) very true/often. They were also given the option to respond with “no opportunity” when caregivers have not had the opportunity to observe their child engaging in particular activities/situations. Raw scores were converted to age-and sex-specific *t*-score (mean = 50, STD = 10). For Externalizing, Internalizing, and Dysregulation domains, a *t*-score ≥ 65 (at or above 90th percentile) is considered “at risk” or “of concern” for social–emotional problems. For Competence domain, a *t*-score ≤ 35 (at or below 10th percentile) is considered “of concern” for a deficit or delay. Trained personnel from the Developing Brain Institute administered the ITSEA to caregivers either in-person or over the phone. Carter et al. (2003) and Briggs-Gowan and Carter (2006) showed that the ITSEA domains exhibited domain specificity, evaluating distinct aspects of socio-emotional development. However, low to moderate associations were also reported between some domains (Carter et al., 2003; Briggs-Gowan and Carter, 2006). To better contextualize our study’s results, we also explored associations between the four domains by calculating Pearson correlations between domain scores.

### 2.3 MRI acquisition

A 3 T scanner (Discovery MR750, GE Healthcare, Milwaukee, WI) was used to acquire structural and functional MR images (referred to sMRI and fMRI, respectively). Structural (sMRI) scanning parameters were as follows: T2-weighted fast spin-echo, TR = 2,500 ms; TE = 64.49 ms, voxel size = 0.625 × 1 × 0.625 mm. For functional MRI (fMRI), resting state scans were acquired using the following parameters: TR = 2000 ms, TE = 35 ms, voxel size = 3.125 × 3.125 × 3 mm, flip angle = 60 degrees, field of view = 200 mm, and matrix size = 64×64. One subject did not have a resting state scan. Scan duration was 6.7 min (200 volumes). Prior to the scan, babies were fed, swaddled in a warm blanket, immobilized using an infant vacuum pillow, and equipped with ear plugs/earmuffs. They were scanned asleep and unsedated. Heart rate and oxygen saturation were monitored by a nurse during the scan.

### 2.4 Preprocessing of MRI data

Structural MRI and fMRI images were preprocessed using AFNI, ANTS, and in-house MATLAB code (Cox, 1996; Avants et al., 2009; De Asis-Cruz et al., 2018). For preprocessing of sMRI, realignment to anterior–posterior commissure, signal intensity normalization, and bias field correction were performed. We then defined the cerebellum into regions of interest using the neonatal SUIT toolbox in SPM (see details in section 2.5) (Hernandez-Castillo et al., 2019). The quality of the automatic cerebellar segmentation was visually examined before analyses.

For fMRI preprocessing, the following steps were applied: (1) within-volume motion correction to fix misaligned slices, (2) correction of slice-time errors, (3) removal of first four volumes to stabilize the magnetization, (4) removal of spikes from the time series (i.e., despiking), (5) bias-field correction, (6) motion correction by registering EPI data to a base volume, (7) co-registration of fMRI into structural MRI template, (8) normalization to a standard neonate brain template using linear and non-linear transformations, (9) intensity normalization (Ojemann et al., 1997), (10) smoothing at 5 mm full-width half-maximum (FWHM), (11) bandpass filtering (0.009–0.08 Hz) (Hallquist et al., 2013), and (12) nuisance regression. For the regression, the nuisance variables were (1) six motion estimates, their first-derivates and quadratic from the rigid-body analysis of head motion (Friston et al., 1996), (2) signals averaged over localized white matter (Jo et al., 2010), and the (3) first three principal components of the ventricular CSF signal (Behzadi et al., 2007; Muschelli et al., 2014). Volumes with excessive head motion (volume-to-volume head motion > 0.2 mm) or >10% of voxels classified as intensity outliers were excluded in the analysis. Voxels comprising the cerebellar hemispheres and the vermis were used in the fMRI analysis. Functional MRI scans with <120 motion-free volumes (=4 min) or corrupted sMRI images were excluded. At the end, the total numbers of sMRI and fMRI scans analyzed in the study were 81 and 72, respectively.

### 2.5 Defining cerebellar features using sMRI and fMRI

**Figure 1:**
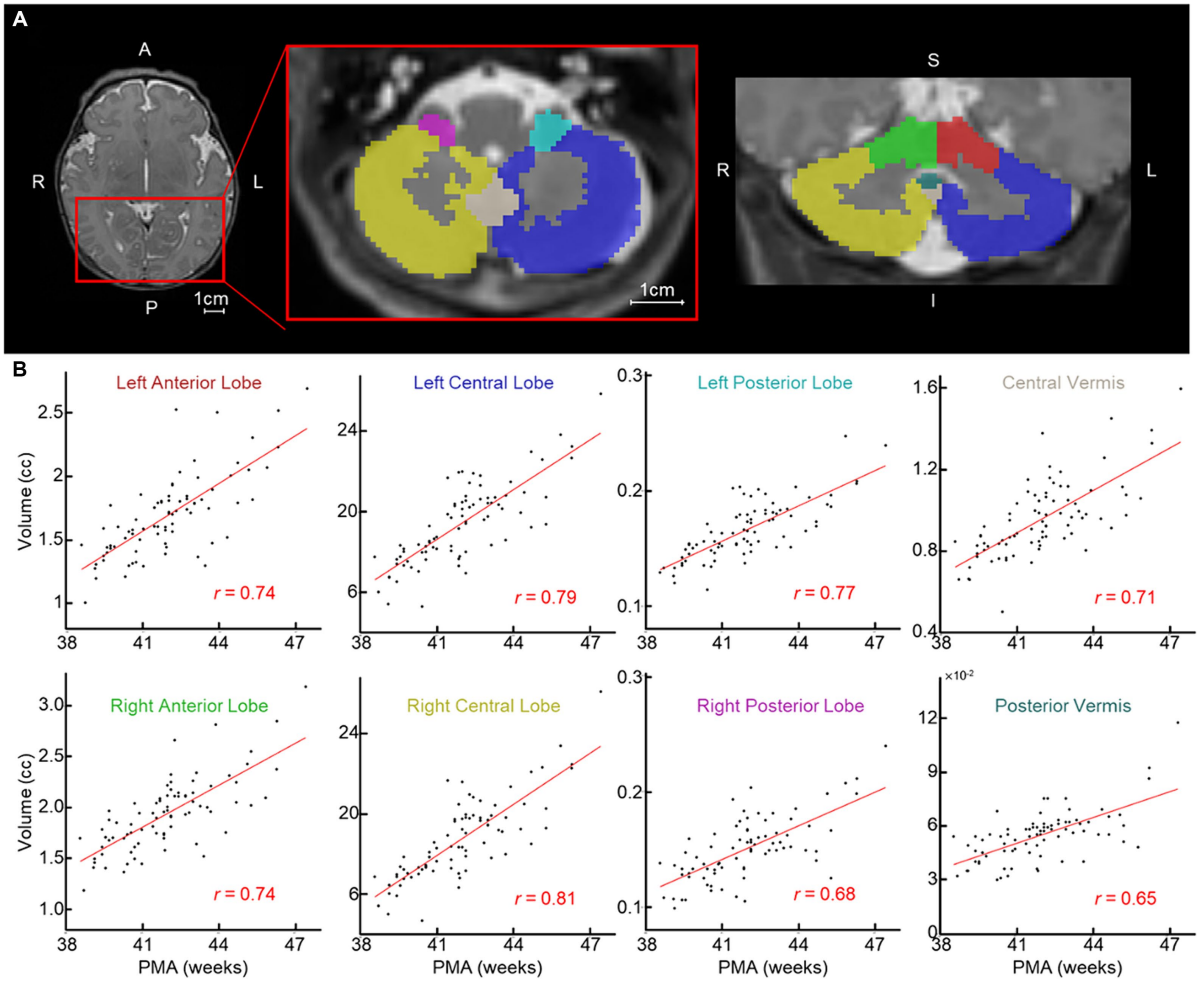
Newborn cerebellar volume increases with PMA. **(A)** Examples of segmented cerebellum (left/right anterior/central/posterior lobes and central/posterior vermis). Each ROI is highlighted with a different color; red and green, left and right anterior lobe; blue and gold, left and right central lobe; teal and pink, left and right posterior lobe; tan and dark teal, central and posterior vermis. **(B)** Scatterplot between PMA (*x*-axis) and volume of cerebellum regions (*n* = 81). Red line = line of best fit. A, anterior; P, posterior; L, left; R, right; S, superior; and I, inferior.

**Figure 2:**
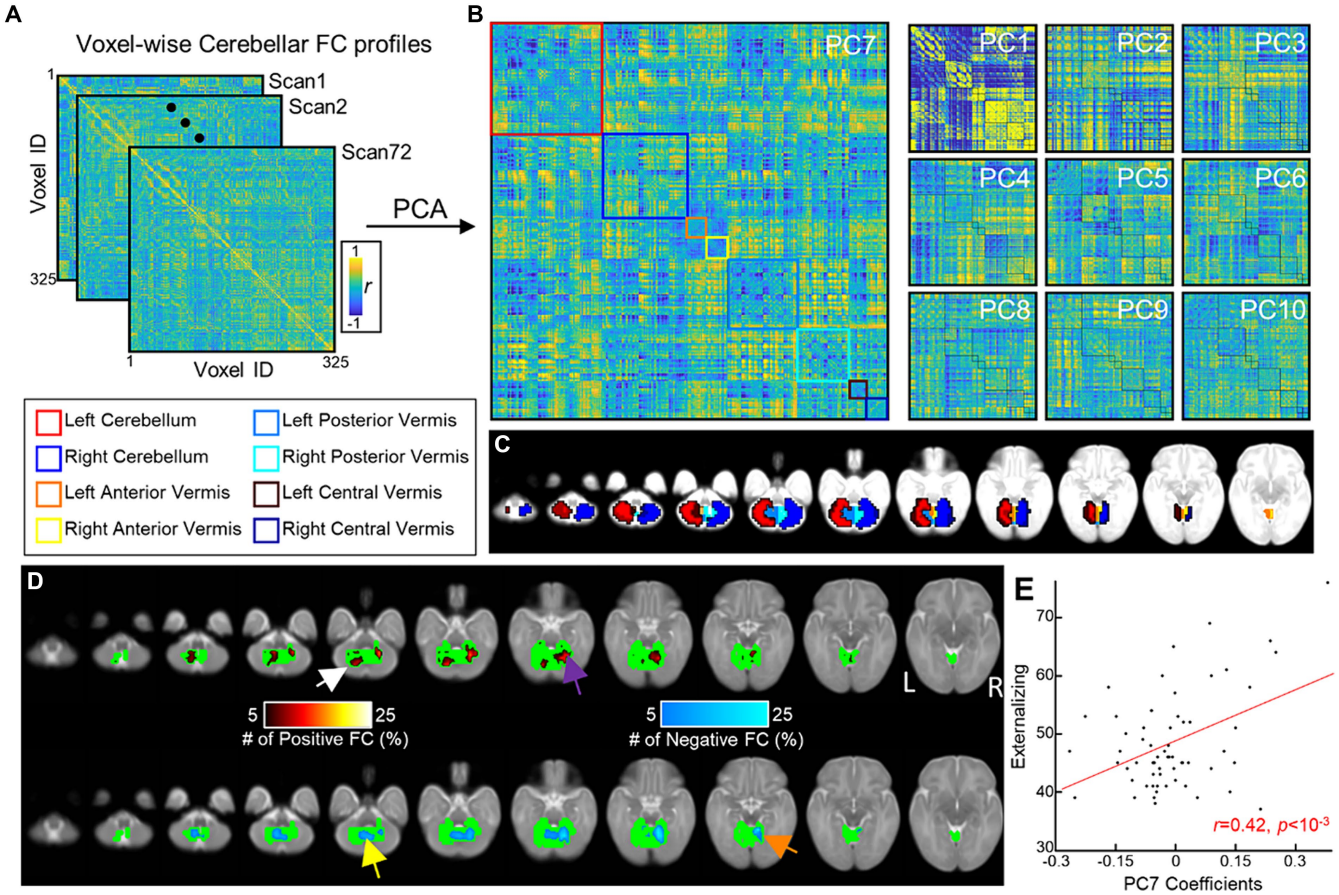
Cerebellar FGRs and their associations with ITSEA scores. **(A)** Voxel-wise cerebellar FC profiles of 72 neonates. **(B)** FGRs of the cerebellum were estimated from **(A)** using PCA. **(C)** Voxels within the cerebellum were assigned to one of cerebellum regions and cerebellar regions were coded as different colors. **(D)** Distribution of top 5% strongest FCs in the 7th principal component (FGR7) (colored using hot/cold color map for positive/negative FCs, respectively). **(E)** Scatterplot between coefficients of FGR7 (*x*-axis) and Externalizing score (*y*-axis). Red line = line of best linear fit.

Based on the segmentation information obtained during sMRI preprocessing, we defined eight cerebellar regions of interest (ROIs: left anterior lobe, right anterior lobe, left central lobe, right central lobe, left posterior lobe, right posterior lobe, central vermis, and posterior vermis) based on the SUIT atlas (Figure 1A). The newborn SUIT divides the cerebellum into 28 ROIs; these were reduced to eight regions to closely approximate the anatomy of the cerebellum described here (Stoodley and Schmahmann, 2018). The areas comprising each of the eight lobes are: left/right anterior lobe — I–IV and V, left/right central lobe/vermis — VI, Crus I, Crus II, VIIb, VIIIa, VIIIb, and IX, and left/right posterior lobe/vermis — X (Supplementary Figure S1). Newborn cerebellar volumes at each of the 8 ROIs served as structural features. Functional features were estimated from the functional gradients of the cerebellum, which was measured as follows. First, for each subject, we estimated voxel-to-voxel functional connectivity (FC) using Pearson correlation. There were 325 cerebellar voxels included in the analysis; these represented areas with consistent signal for all newborns (see green area in Supplementary Figure S2 for the location of these voxels). Thus, each subject had a 325 × 325 voxel-wise cerebellar functional connectivity matrix. The upper triangle of each subject’s matrix was vectorized and analyzed at the group-level (size: 52650 × 72; # of connections × # of subjects). We applied principal component analysis (PCA) to the group-level cerebellar FC. The functional gradients (FGR) were defined by the loading of the top-10 principal components (=52,650 × 10). Here, the number of components (=10) was chosen heuristically based on the observation that the top-10 principal components collectively explained nearly half of total variance (=48.7%). The FGR coefficients (=10 × 72) served as functional features of the cerebellum for each subject (Figures 2A,B).

### 2.6 Assessing associations between ITSEA scores and cerebellar structure and function

The association between scores on each ITSEA domain and cerebellar volume was tested using a linear regression model (*fitlm.m* in MATLAB R2020a). Totally, 71 subjects having both ITSEA scores and cerebellar volume were included in the analysis. A separate linear model was used for each domain. Model covariates included biologic sex, birthweight, and postmenstrual age (PMA), variables that may confound the estimated relationship between domain scores and brain features. Apgar scores were not included as covariates as most were between 8 and 9. The associations between scores on the ITSEA subscales and functional cerebellar features (i.e., FGR) were investigated in the same manner (# of subjects = 62). False discovery rate (FDR) was used to correct for multiple comparisons and was performed using the MATLAB function *fdr_bh.m*.^1^ The latter function implements the original FDR correction described here (Benjamini and Hochberg, 1995).

### 2.7 ITSEA score prediction using multimodal cerebellar features

In the previous analysis, we observed that certain cerebellar features were associated with ITSEA scores at 18 months. To reinforce this association, we additionally predicted ITSEA scores from anatomical/functional MRI features. To test whether anatomical and functional cerebellar features were complementary to each other; we performed the prediction task by combining two feature sets and compared it to one obtained from single feature set, structural or functional. Of the 88 subjects, only the 61 with available ITSEA scores, sMRI, and fMRI scans were included in the prediction task. We used a 10-fold cross-validated linear regression model to predict the score for each ITSEA domain (i.e., one prediction model for each domain) using cerebellar features. As this prediction task was performed under the cross-validation scheme, non-imaging predictors (such as PMA, sex, and birth weight) were not included in the prediction model. We used three different feature sets: (1) a combination of anatomical and functional cerebellar features, (2) only functional features, and (3) only anatomical features. For the functional feature, for each fold, we re-evaluated the FGR (see above section 2.5 for the detailed methods of FGR). The PCA for calculating FGR was applied within the training dataset, to prevent potentially introducing bias to the prediction task. Then, the FGR coefficients of the training dataset were used for training the prediction model. Using the held-out (i.e., test) dataset, we evaluated the prediction performance of the trained model. The prediction performance was evaluated by estimating the correlation between predicted and actual ITSEA scores.

The ability of cerebellar volume and FGR to predict the ITSEA domain score was assessed using permutation testing. The distribution of null prediction performance was generated from 5,000 permutation tests. For each test, we randomly shuffled ITSEA scores of the 61 subjects and calculated prediction performance based on the shuffled scores. A *p*-value was calculated based on the proportion of permuted statistics as extreme as or more extreme than the actual or observed prediction performance. If the *p*-value was below the significance threshold (i.e., 0.05), the result was considered statistically significant. Permutation test was implemented using in-house MATLAB code.

## 3 Results

### 3.1 Subjects

We evaluated structural and functional MRI data from 88 healthy newborns (postmenstrual age/PMA in weeks, mean ± sd of = 42.00 ± 1.91; range = 38.57–47.43; 43 M/45F). Among 88 subjects, 73 were scanned during the neonatal period (<4 weeks of life); the rest (=15) were scanned before 48 weeks. Table 1 shows demographic information for all subjects.

**Table 1:** Demographic information of neonate cohort.

Demographics (*n* = 88)	Mean (*SD*)	Range
GA at birth (weeks)	39.42 (1.14)	37.14–41.57
PMA at scan (weeks)	42.00 (1.91)	38.57–47.43
# of male/female	43/45	
Birth weight (g)	3,303 (432)	2,280–4,184
# of AGA, SGA, LGA	74, 12, 2	
Apgar 1 (median; IQR 25|75)	8; 8|9	2–10
Apgar 5 (median; IQR 25|75)	9; 8|9	5–10

SD, standard deviation; PMA, post-menstrual age; GA, gestational age. AGA, SGA, and LGA, appropriate, small, and large for GA. Apgar 1 and 5 not available for 2 subjects.

Of the 88 neonates, 77 (88%) completed ITSEA assessments during toddlerhood (mean age on ITSEA: 18.32 ± 1.19 months old). Among the 77, only a very small number – one to four subjects – had scores categorized as “of concern.” The breakdown of the ITSEA scores for our cohort is provided in Table 2. The demographic information for neonates with ITSEA scores deemed “of concern” is provided in Table 3. The correlations between domains observed in our cohort closely mirrored that of the original ITSEA study (Carter et al., 2003; Briggs-Gowan and Carter, 2006). We found significant associations between the following domains: Externalizing-Internalizing (*r* = 0.26, *p* = 0.030), Externalizing-Dysregulation (*r* = 0.29, *p* = 0.015), and Internalizing-Dysregulation (*r* = 0.39, *p* = 0.001). We did not observe a significant correlation between Dysregulation and Competence domains (*r* = −0.21, *p* = 0.078); a negative association was previously reported by (Carter et al., 2003). Lastly, the Competence domain was not associated with any other domains (*r* = −0.00, *p* = 0.978 for Externalizing and *r* = −0.05, *p* = 0.680 for Internalizing).

**Table 2:** Summary of newborn ITSEA scores.

*t*-score	ITSEA score (*n* = 77)			
	Externalizing	Internalizing	Dysregulation	Competence
Mean	48.4	46.0	40.5	50.5
Median	46	44	39	51
Standard deviation	7.8	8.2	9.0	9.5
# of “of concern” (criteria)	4 ( ≥ 65)	1 ( ≥ 65)	1 ( ≥ 65)	4 ( ≤ 35)

**Table 3:** Demographic information of high-risk neonate cohort.

Mean (*SD*)	Externalizing (*n* = 4)	Internalizing (*n* = 1)	Dysregulation (*n* = 1)	Competence (*n* = 4)
GA at birth (wks)	39.00 (0.72)	37.29	40	38.96 (0.71)
PMA at scan (wks)	40.97 (1.02)	39.71	41.9	41.03 (2.31)
# of male/female	2/2	0/1	1/0	2/2
Birth weight (g)	3,078 (344)	3,350	2,990	3,515 (232)
# of AGA, SGA, LGA	3/1/0	1/0/0	0/1/0	4/0/0
Apgar 1 (median)	8	10	9	9
Apgar 5 (median)	9	10	9	9

SD, standard deviation; PMA, post-menstrual age; GA, gestational age. AGA, SGA, and LGA, appropriate, small, and large for GA.

### 3.2 Volume of the right posterior cerebellar lobe is associated with the internalizing domain

The volumes of all eight cerebellar ROIs increased with advancing PMA (Figure 1B; range of *r* = 0.65–0.81; FDR-corrected *p* < 10^−7^ for all). PMA at scan accounted for much of the increase in volume of the cerebellum, explaining on average 54.57% of the variance (min: 41.85% from posterior vermis, max: 65.53% from right central lobe). We then assessed whether newborn cerebellar volume was related to ITSEA scores at around 18 months. The volumes of the cerebellar ROIs were not significantly correlated with scores on the ITSEA domains (Table 4). The volume of the right posterior lobe tended to decrease with increasing Internalizing scores, but this finding did not survive multiple comparison correction (*t*-score = −2.01, uncorrected *p* or *p_unc_* = 0.049; Supplementary Figure S3).

**Table 4:** Association between cerebellum volume and ITSEA scores.

Brain region	ITSEA score ~ Volume + Sex + PMA + birth weight *t*-score (*p_unc_*-value); *n* = 71			
	Externalizing	Internalizing	Dysregulation	Competence
Anterior lobe, L	−0.52 (0.605)	−0.72 (0.472)	−1.52 (0.132)	−1.14 (0.257)
Anterior lobe, R	−0.34 (0.735)	−1.20 (0.236)	−1.94 (0.057)	−1.28 (0.206)
Central lobe, L	0.30 (0.762)	0.80 (0.426)	0.01 (0.994)	0.20 (0.839)
Central lobe, R	−0.49 (0.626)	0.41 (0.686)	−0.33 (0.739)	0.13 (0.897)
Posterior lobe, L	0.52 (0.605)	−0.27 (0.792)	0.44 (0.665)	−1.02 (0.310)
Posterior lobe, R	0.18 (0.854)	−2.00 (0.049)^*^	−1.19 (0.239)	−0.48 (0.632)
Central vermis	0.02 (0.985)	1.27 (0.209)	−0.18 (0.858)	0.40 (0.692)
Posterior vermis	−0.26 (0.796)	0.12 (0.906)	0.06 (0.955)	−0.00 (0.997)

^*^Uncorrected *p* < 0.05.

### 3.3 Cerebellar functional gradient is associated with externalizing, internalizing, and competence scores

The FGR patterns were identified using PCA (Figures 2A,B). Of the 71 FGRs (=# of subjects −1), we selected the top 10 (Figure 2B) that explained 48.67% or about half of the variance in the voxel-wise FC profiles. Specifically, each of the 10 FGRs accounted for the following: FGR1 = 28.86, FGR2 = 3.13, FGR3 = 2.68, FGR4 = 2.50, FGR5 = 2.31, FGR6 = 2.04, FGR7 = 2.00, FGR8 = 1.80, FGR9 = 1.72, and FGR10 = 1.61. The pattern of FGR1 showed strong correlations and anti-correlations (*r* values close to 1 or −1) possibly reflecting similarity (dissimilarity) in FC profiles among neighboring (distant) voxels.

We found no significant associations between FGRs and PMA (min and max of *r* = −0.12 and 0.19 and *p* = 0.31 and 0.11, for FGR2 and FGR9). We then investigated whether the FGR coefficients were associated with newborn social–emotional development (Table 5). The coefficients of FGR7 were significantly associated with the Externalizing domain (Externalizing: *t*-score = 3.39, *p_unc_* = 0.001 and *p_FDR_* = 0.013). There were also trending associations (*p_unc_* < 0.05) between FGR7-Internalizing score (*t*-score = 2.19; *p_unc_* = 0.032 and *p_FDR_* = 0.32), FGR1-Competence score (t-score = 2.50; *p_unc_* = 0.015 and *p_FDR_* = 0.15), and FGR10-Competence (*t*-score = −2.08, *p_unc_* = 0.042 and *p_FDR_* = 0.21). The Dysregulation domain was not significantly associated with any of the FGRs. The top 5% of the strongest positive (hot/red) or negative (cold/blue) connectivity values comprising FGR7 localized into four clusters (Figure 2D; each cluster highlighted with an arrow). These are the: (1) left dentate (white arrow), (2) right lobule VI (purple arrow), (3) left vermis VIIIb (yellow arrow), and (4) right lobule V (orange arrow). Note that there was no significant effect of sex. Correlation analysis showed that neonates with higher FGR7 coefficients tended to have higher Externalizing score (Figure 2E; *r* = 0.42, *p* < 10^−3^).

**Table 5:** Association between cerebellar FGRs and ITSEA scores.

Cerebellar FC	Score ~ FC + PMA + Sex + Birth weight *t*-score (*p_unc_*-value); *n* = 62			
	Externalizing	Internalizing	Dysregulation	Competence
FGR1	1.21 (0.230)	0.37 (0.713)	0.93 (0.357)	2.50 (0.015)^*^
FGR2	−1.30 (0.200)	0.78 (0.442)	−0.40 (0.689)	0.73 (0.468)
FGR3	−1.47 (0.146)	−0.26 (0.796)	1.99 (0.051)	−1.17 (0.248)
FGR4	1.18 (0.241)	0.11 (0.912)	−0.26 (0.794)	−0.61 (0.544)
FGR5	−0.35 (0.728)	−0.19 (0.852)	0.21 (0.838)	0.01 (0.996)
FGR6	0.16 (0.872)	1.43 (0.157)	0.59 (0.559)	1.40 (0.167)
FGR7	3.39 (0.001)^**^	2.19 (0.032)^*^	1.19 (0.238)	0.74 (0.464)
FGR8	0.05 (0.962)	−0.08 (0.936)	0.91 (0.368)	1.14 (0.258)
FGR9	0.77 (0.447)	0.66 (0.514)	−0.55 (0.588)	0.62 (0.539)
FGR10	−1.22 (0.226)	1.11 (0.273)	1.17 (0.245)	−2.08 (0.042)^*^

^*^Uncorrected *p* < 0.05, ^**^FDR-corrected *p* < 0.01.

### 3.4 Prediction of ITSEA scores using anatomical and functional features of the cerebellum

**Figure 3:**
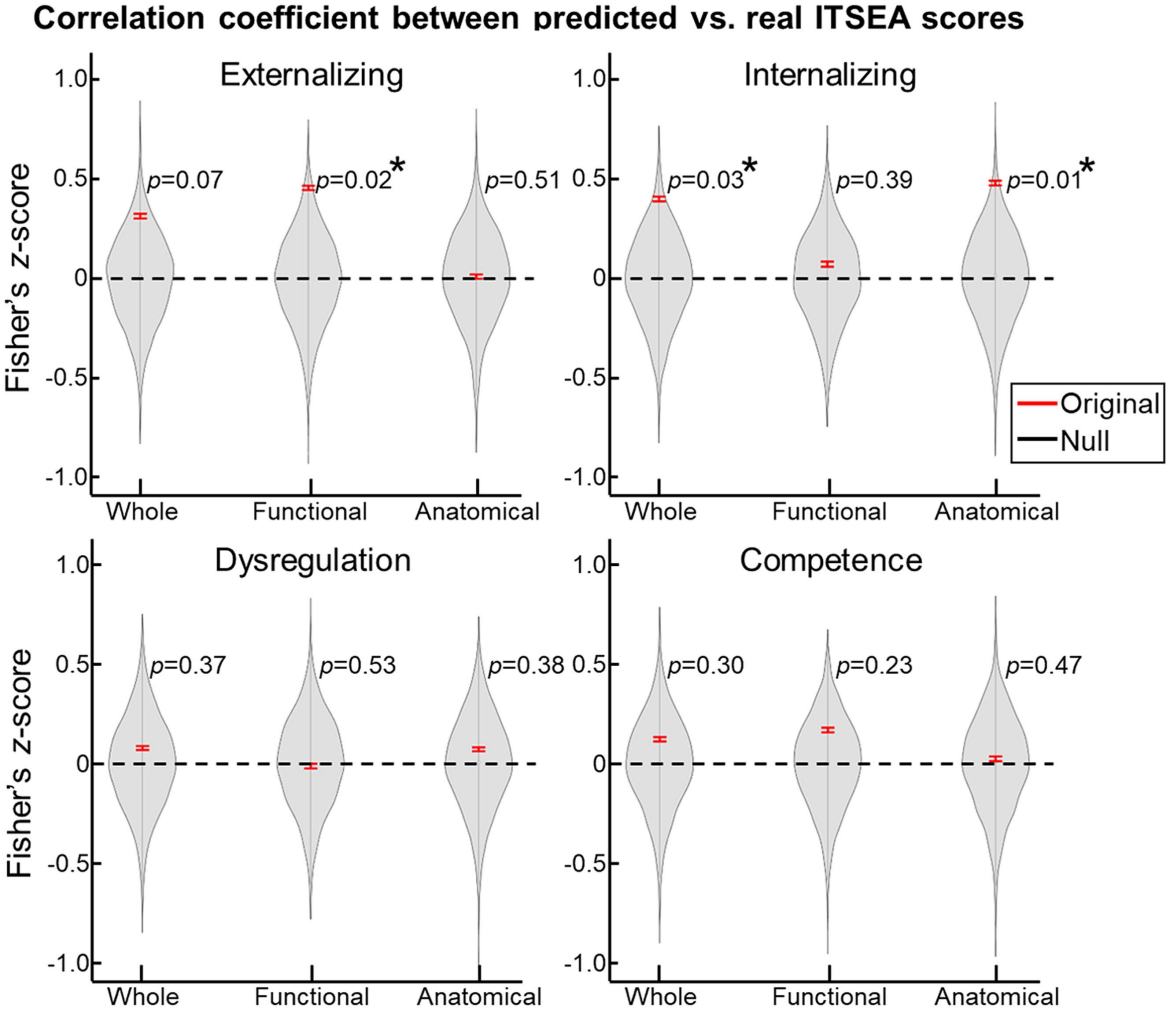
Prediction of ITSEA scores using cerebellar anatomical and functional features. Using 10-fold cross validation, we built a linear regression model that predicted ITSEA scores from functional, anatomical, or both sets of features. Prediction performance is evaluated by estimating the correlation between actual and predicted scores (**left top**: Externalizing, **right top**: Internalizing, **left bottom**: Dysregulation, and **right bottom**: Competence) in the test dataset (red error bar). Error bars depict standard error of the mean. Violin plots represent the null distribution of prediction accuracies generated by permutation testing. *FDR-corrected *p* < 0.05.

**Figure 4:**
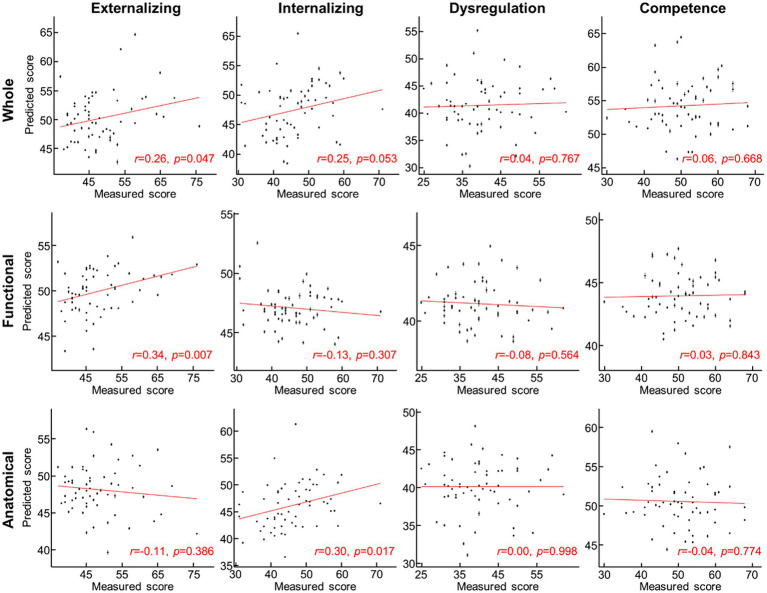
Scatterplot between measured and predicted ITSEA scores. Predicted ITSEA scores (columns) were estimated from functional, anatomical, or both sets of features of the cerebellum (rows). Black dot = single subject data; error bar = standard deviation across 100 prediction trials with different 10-fold allocation of subjects. Red line = line of best fit.

For the prediction task, we built a 10-fold cross-validation linear regression model (i.e., one model for each domain) that predicted the ITSEA domain score from anatomical, functional, or both anatomical and functional features of the cerebellum. Externalizing and Internalizing scores were significantly predicted by anatomical and/or functional features of cerebellum (Figure 3). Anatomical features (i.e., volume of the subdivisions of the cerebellum) predicted the Internalizing score (mean ± S.E of Fisher’s *z* = 0.48 ± 0.01 vs. 0.00 ± 0.00 with permutation, *p* = 0.01; real>permutation). Functional features predicted scores on the Externalizing domain (mean ± S.E of Fisher’ *z* = 0.45 ± 0.01, *p* = 0.02). The prediction model using combined anatomical and functional features showed lower prediction performance compared to the model with a single feature set. Predicted Externalizing (using functional features) and Internalizing (using anatomical features) scores averaged over 100 repeated cross-validated prediction tasks were significantly correlated with the actual scores (Figure 4; *r* = 0.34, *p* = 0.007 for Externalizing-functional feature; *r* = 0.30, *p* = 0.017 for Internalizing-anatomical feature). All the associations between ITSEA scores and feature sets can be found in Figure 4. The findings from the prediction task reinforced the regression model findings and suggested that newborn cerebellar anatomy and function could forecast social and emotional wellbeing in early childhood. Importantly, we found that specific domains were closely associated with different anatomical and functional features: the Externalizing domain associated with functional features and the Internalizing domain associated with anatomical feature.

## 4 Discussion

We investigated whether regional cerebellar volume and connectivity in newborns obtained using multimodal MRI were associated with social–emotional development evaluated using the ITSEA at 18-months. We found that cerebellar volume was not associated with scores on any of the ITSEA domains (Table 4), while functional gradient was significantly correlated with the Externalizing domain (Figure 2 and Table 5). One functional gradient, FGR7, comprised of four clusters that included the left dentate, right VI, left Vermis VIIIb, and right V lobules explained the largest variance in Externalizing score (Figure 2D). Furthermore, we successfully predicted two domains (Externalizing and Internalizing) of the ITSEA using either the structural or functional features of the cerebellum (Figures 3, 4). These findings emphasize the critical role of the cerebellum in early social–emotional development and highlight the specific contributions of cerebellar structure and function to toddler temperament.

The social role of the cerebellum in healthy human adults has been previously described [see Hoche et al. (2016), Sokolov (2018), Van Overwalle et al. (2019), Van Overwalle et al. (2020, 2021), Olson et al. (2023)]. One meta-analysis has reported consistent engagement of the posterior areas of the cerebellum (e.g., VIIB, VI, Crus I, IV, VI, and IX) in social tasks (Van Overwalle et al., 2014). One clinical study has also reported enlarged Crus I and II volume in patients with impaired emotional awareness or alexithymia (Petrosini et al., 2015). Much less is known about the associations between socio-emotional behavior and cerebellar structure and function in typically developing neonates. But, clinical studies have noted delayed social and emotional development in toddlers with prenatal cerebellar injury (Steinlin et al., 1998; Limperopoulos et al., 2007; Tavano et al., 2007; Parker et al., 2008; Steggerda et al., 2009; Hayashi et al., 2015; Bulgheroni et al., 2016; Hickey et al., 2018). As cerebellar injury often impacts multiple regions or a large portion of the cerebellum, it is challenging to isolate the specific social role of each cerebellar lobule in neonates. Notably, in children aged 1–6 years-old, malformations in different cerebellar subregions have been associated with developmental delays (Pinchefsky et al., 2019). Additionally, it has been demonstrated that full-term newborns already exhibit a functional topography of the cerebellum, similar to that seen in adults (Herzmann et al., 2019). Similar functional segregation of cerebellum was observed in our study; four clusters of functional gradient (FGR7) locating in four cerebellar lobules (left dentate, right VI, left Vermis VIIIb, and right V; highlighted by arrows in different colors in Figure 2). This gradient was significantly associated with Externalizing behavioral trait in typically developing infants (Table 5). Notably, these four cerebellar lobules are part of the posterior cerebellum, which aligns with findings in human adults (Habas et al., 2009; Krienen and Buckner, 2009; O'reilly et al., 2010). Findings from our study and others (Herzmann et al., 2019) collectively suggest that the functional segregation of cerebellum begins as early as the neonatal period.

Interestingly, in our normative cohort, we observed a negative relationship between lower Internalizing scores (indicating lower risk of Internalizing problems) and enlarged volume of the right posterior lobe (Supplementary Figure S3 and Table 4). This finding did not survive FDR correction, however, we note it here because this negative association aligns with previous findings in children and adolescents (Laidi et al., 2015; Lin et al., 2020). Past studies in older children have reported links between Externalizing scores and cerebellar volume in healthy older children (Borges et al., 2022), adolescents (Jarvers et al., 2022), and adults (Hill et al., 2016); we did not observe this relationship in our cohort. We speculate that the discrepancy in the association between cerebellar volume and Externalizing domain in the neonate compared to findings in other age groups was, at least partially, due to two factors: (1) different granularity levels of defined cerebellar subregions and (2) utilization of different social–emotional measures. Regarding (1), due to several practical reasons such as lower image resolution, smaller cerebellum size, and under-defined cerebellar lobules in neonates, cerebellum was clustered into eight subregions vs. finer subregions in other age groups. For the latter, in our study, the externalizing aspect of subjects was quantified using ITSEA, which differs from ones used in age groups, e.g., Child Behavior Checklist for children and Strength and Difficulties Questionnaire for adolescents. We can at least rule out two other possible explanations; inaccurate measurement of cerebellar volume or failure of ITSEA score as a representation of social–emotional traits of neonates. First, we believe the lack of association in our study was unlikely from measurement errors in cerebellar volume. This is because we were able to observe a significant trend between cerebellum volume growth and PMA (Figure 1). Second, if the ITSEA scores obtained at 18 months were not a reliable representation of the social–emotional traits of neonates, the functional features of the cerebellum would also fail to predict the Externalizing domain, which was not the case (Figure 3). Hence, we believe the second possibility was unlikely too. Due to limitations such as the smaller size of the neonatal cerebellum, lower spatial resolution, poorer tissue contrast in neonatal MRI, and poorer definition of cerebellar lobules, we were unable to reliably segment each subject’s cerebellum into lobules. However, based on the association of certain personality traits with specific cerebellar lobules in human adults (e.g., Crus I and II) (Petrosini et al., 2015), we posit that the volume of specific cerebellar lobules, particularly in the posterior part, may predict a broader range of social–emotional traits in neonates. We hope that future research will confirm our speculation.

We provided novel insights into the relationship between multimodal features of the cerebellum and social–emotional development in a normative neonatal cohort. We observed that the structural and functional features of the cerebellum predicted different social–emotional domains: functional gradients predicted Externalizing domain and cerebellar volume predicted Internalizing score (Figure 3). It is noteworthy that our observations in the prediction task were largely in line with findings from the association analysis (Tables 4, 5), further supporting that the observations in this study were likely driven by neurobiological factors. However, the relationship between cerebellar volume and the Internalizing domain was inconsistent. Volume significantly predicted Internalizing score, but there was no significant correlation between volume and Internalizing score after applying multiple comparison correction. This discrepancy may be attributed to the use of different statistical methods in the two tasks: conventional multiple comparison correction (e.g., false-discovery-rate correction) in the analytical test versus a permutation scheme in the prediction task. Future studies are required to validate the origins of the disparity between prediction and correlation analyses. In addition, distinct social–emotional roles between functional-and structural features of neonatal cerebellum were somewhat inconsistent with observations in adults, reporting functional and structural roles of the cerebellum in mentalizing (Metoki et al., 2022). One possible explanation for discrepancies in the findings across these studies is that our functional features were defined using cerebellar connectivity only while functional features in the above study were based off cerebro-cerebellar connectivity. Our findings may suggest the possibility that within-cerebellum connectivity can provide unique social–emotional aspects of cerebellum that differ from global cerebro-cerebellar connectivity. Another consideration is that the neurobehavioral role of the maturing cerebellum may differ from that of the matured cerebellum. In line with this perspective, a recent study proposed a hypothesis suggesting that the human brain constructs a social-mental model in the cerebellum during early life as a “forward model,” which is subsequently utilized in later stages as an “inverse model” (Olson et al., 2023). Although this hypothesis requires further investigation, it offers a potential explanation for the observed differences.

Externalizing disorders can be characterized by behaviors such as inattention, hyperactivity, aggression, substance use, or rule-breaking (Cosgrove et al., 2011), and internalizing disorders can be characterized by anxiety, depressive, and somatic symptoms (Andersen and Bienvenu, 2011). Both can be commonly observed in patients with various neuropsychiatric disorders (Eaton et al., 2013). While most of our cohort’s Externalizing and Internalizing ITSEA scores fell within the normal range, our results suggest that newborn cerebellar structural and functional signatures may serve as potential biomarkers of later social–emotional impairments, especially given the involvement of the cerebellum in various neuropsychiatric disorders, such as schizophrenia (Yeganeh-Doost et al., 2011), ADHD (Silk et al., 2009), major depression (Fitzgerald et al., 2008), bipolar (Baldacara et al., 2011), and autism (Bauman and Kemper, 2003).

Our study has several limitations. First, that majority of our subjects fell within the normative range of ITSEA social–emotional scores. To fully characterize brain-behavior associations, inclusion of more “at risk” individuals is needed. Our regression and prediction task suggested the scores can be reliably explained or predicted by cerebellar features but whether “at risk” subjects can be identified based on the features needs to be investigated further. Recruiting more healthy neonates with social–emotional developmental concern, defined by beyond “at risk” ITSEA scores, is needed to understand the social–emotional role of cerebellum more deeply. Second, due to several factors such as lower spatial resolution, smaller brain size, weaker tissue contrast, and under-defined brain lobules in neonates compared to human adults, we encountered challenges in reliably segmenting the cerebellum into distinct lobules for each neonate, as discussed earlier. However, previous studies involving human adults have suggested that each cerebellar lobule is responsible for specific aspects of motor, cognitive, and social–emotional function. Therefore, we propose that incorporating lobule-specific structural features may improve prediction of social–emotional development in neonates. Third, it is uncertain whether our findings will be generalizable across different datasets. To our knowledge, there are no publicly available datasets having sMRI and fMRI of neonates and follow-up social–emotional surveys. Future validation of our findings in various datasets with different MRI acquisition parameters and preprocessing steps will be needed to confirm our findings in social–emotional role of cerebellum in the developing brain. Furthermore, all neonatal scans were conducted during natural sleep, a widely adopted approach in newborns due to challenges with controlling head motion and monitoring wakefulness during this period. The impact of sleep (and its various stages) on functional connectivity in the developing brain, however, remains to be fully explored. Lastly, while our findings align with the current literature, interpretation of the FGRs themselves as well as the association between FGRs and neurologic outcomes remain somewhat limited. Prenatal MR imaging is in its early stages, and researchers in the field are just beginning to explore the neurobiological bases of rs-fMRI in newborns. In adults, various biophysical signals, including low-frequency fluctuations in cardiac rate (Shmueli et al., 2007), arousal level (Chang et al., 2016), and postsynaptic activity (Mantini et al., 2007), have been identified as sources of intrinsic brain fluctuations. It remains to be seen which signals significantly contribute to the newborn BOLD response.

Based on the findings of our study, we would like to highlight some promising directions for future research. First, while our study focused primarily on the cerebellum, a substantial body of literature in human adults has emphasized the importance of cerebro-cerebellar functional connectivity in social–emotional functioning (Metoki et al., 2022; Wu et al., 2022). Thus, expanding the investigation to include functional features spanning the entire brain, including the cerebral cortex, hippocampus, and thalamus, can offer a more comprehensive understanding of the cerebellum’s role in social–emotional processes during the neonatal stage. Second, incorporating a broader range of morphological features can provide further insights into the intricate relationship between the structural characteristics of the cerebellum and the social–emotional development of neonates. By examining additional morphological markers and their associations with specific domains of social–emotional functioning, we can gain a more detailed understanding of the complex interplay between cerebellar structure and neonatal development. These future directions hold significant potential for advancing our knowledge base of the cerebellum’s involvement in social–emotional development during early critical periods.

## 5 Conclusion

For the first time, we demonstrated that multimodal MRI data capturing the structural and functional characteristics of the neonatal cerebellum are significantly associated with their social–emotional development at 18 months of age. Specifically, we found a significant correlation between the functional feature, represented by the functional gradients obtained through PCA, and the Externalizing trait. Importantly, we demonstrated the robust predictive power of both structural and functional features for neonatal social–emotional traits. These findings shed new light on the critical role of the cerebellum during the neonatal stage in facilitating healthy social–emotional development, and they hold promise for the development of early biomarkers for childhood and adult neuropsychiatric disorders.

## Data availability statement

The raw data supporting the conclusions of this article will be made available by the authors, without undue reservation.

## Ethics statement

The studies involving humans were approved by Institutional Review Board (IRB) of Children’s National. The studies were conducted in accordance with the local legislation and institutional requirements. Written informed consent for participation in this study was provided by the participants’ legal guardians/next of kin.

## Author contributions

J-HK: Conceptualization, Data curation, Investigation, Methodology, Resources, Software, Validation, Visualization, Writing – original draft, Writing – review & editing. KK: Methodology, Writing – review & editing. CL: Conceptualization, Funding acquisition, Supervision, Validation, Writing – review & editing. JA-C: Conceptualization, Methodology, Supervision, Validation, Writing – review & editing.

## Footnotes

^1^https://www.mathworks.com/matlabcentral/fileexchange/27418-fdr_bh
