# Supplementary material for: Cerebellar volume and functional connectivity in neonates predicts social and emotional development in toddlers
Source: Front Neurosci. 2024 May 1;18:1294527. doi: 10.3389/fnins.2024.1294527 (PMC11097671; doi:10.3389/fnins.2024.1294527)
Supplement: Supplementary file 1 [file Data_Sheet_1.docx]

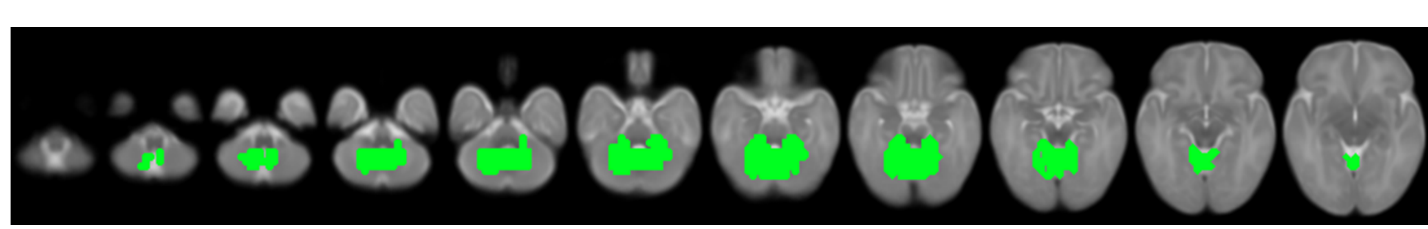


**Supplementary Figure 1. Cerebeller voxels included in the analysis.**


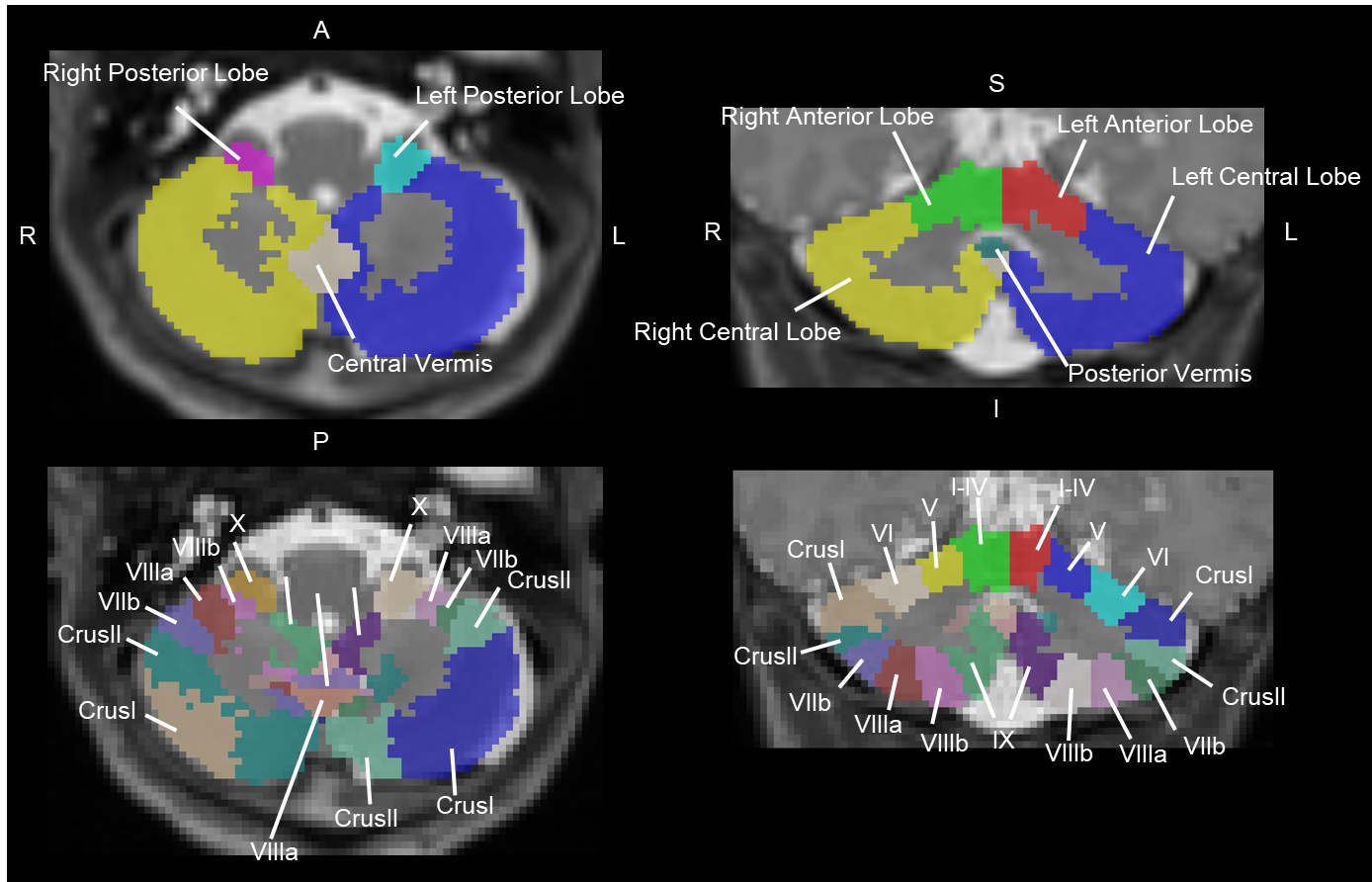


**Supplementary Figure 2. Regions of interest in SUIT cerebellum template.**


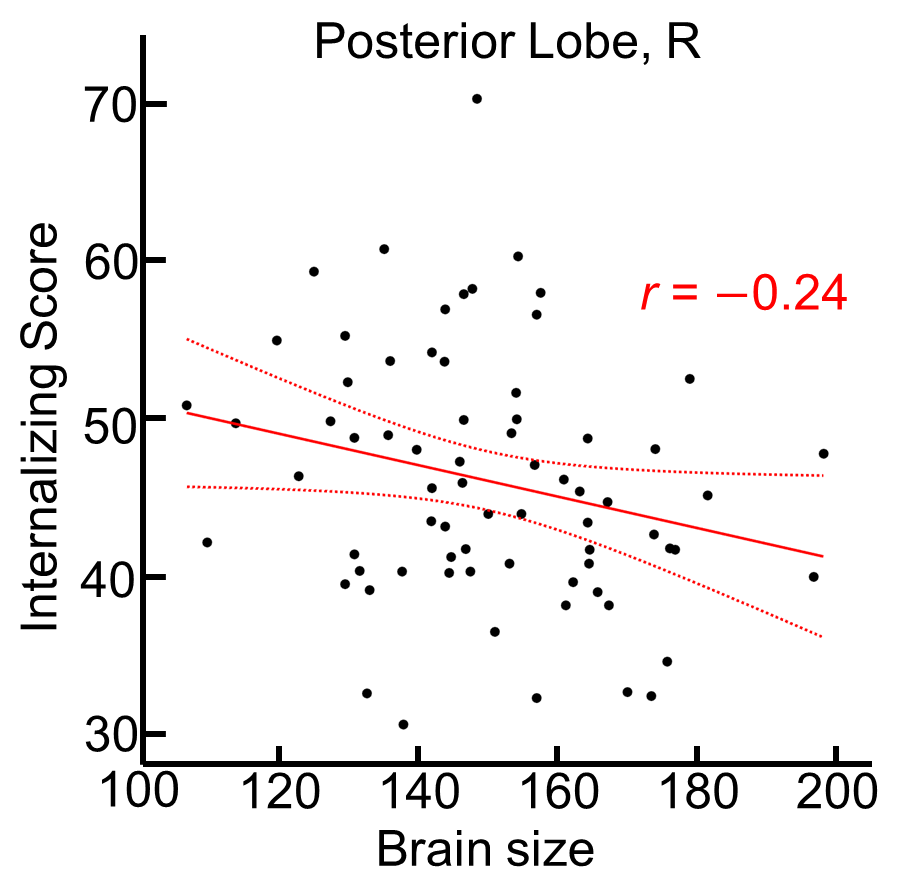


**Supplementary Figure 3. Association between newborn right posterior lobe volume and Internalizing Score of the ITSEA.** The ITSEA Internalizing score (y-axis) was regressed on the volume of right posterior cerebellar lobe (x-axis) and other co-variates: biologic sex, postmenstrual age (PMA), and birth weight (BW). Red solid and dashed line represent line of best fit and 95% of confidence interval, respectively. n= 71**.**
